# Association of dietary fiber intake with metabolic syndrome among adult cancer survivors: a population-based cross-sectional study

**DOI:** 10.1038/s41598-021-91312-1

**Published:** 2021-06-03

**Authors:** Kyuwoong Kim, Yoonjung Chang

**Affiliations:** 1 Division of Cancer Control and Policy, National Cancer Control Institute, National Cancer Center, 323 Ilsan-ro, Ilsandong-gu, Goyang, Gyeonggi-do 10408 Republic of Korea; 2 Department of Cancer Control and Population Health, Graduate School of Cancer Science and Policy, National Cancer Center, Goyang, Republic of Korea

**Keywords:** Epidemiology, Outcomes research, Nutrition

## Abstract

Nutrient intake for adult cancer survivors is of clinical importance for managing metabolic health. Whether dietary fiber intake is associated with metabolic syndrome (MetS) or not in adult cancer survivors is uncertain. We aim to investigate the association between dietary fiber intake and MetS in adult cancer survivors using a population-based cross-sectional study. A study sample of 1301 adult cancer survivors aged more than 20 years from the sixth and seventh Korea Nutrition Examination Survey (KNHANES) from 2013 to 2018 was identified. Odds ratio (OR) and 95% confidence intervals (95% CI) were estimated from multiple logistic regression adjusted for sociodemographic factors, health behavior, and nutritional status. Among 1,301 adult cancer survivors identified from the KNHANES 2013–2018, the mean dietary fiber intake was 28.1 g/day (standard error, 0.54). Compared to the first quintile of dietary fiber intake, the adjusted ORs and 95% CIs for MetS in the second, third, fourth, and fifth quintiles of dietary fiber intake were 0.84 (0.27–2.61), 0.77 (0.16–3.74), 0.55 (0.14–2.22), and 0.26 (0.05–1.39), respectively (*p* value for trend = 0.0007). Our findings suggest that high dietary fiber intake is marginally associated with reduced odds of MetS in adult cancer survivors.

## Introduction

*Nutrition and Physical Activity Guidelines for Cancer Survivors* were released by the American Cancer Survivors (ACS) in 2012^1^. The ACS recommends that cancer survivors take sufficient dietary fiber as a part of healthy and balanced diet such as lower proportion of dietary saturated fat with emphasis on vegetables and fruits intake for improved health outcomes^2–4^. Based on the most recent meta-analysis of cross-sectional and cohort studies reported in 2018, high dietary intake of fiber is inversely associated with metabolic syndrome (MetS) in the general population^5,6^. The accumulated evidence shows limited association and dose–response relationship between dietary fiber intake and MetS, potentially owning to the limited data from cohort studies. Furthermore, on the basis of the currently available guidelines and evidence, association between dietary fiber intake and MetS has not been clearly established, especially in the cancer survivors whose potential health benefits from dietary fiber is of public health importance for policy to support cancer survivors^7–10^.

High intake of dietary fiber has been inversely associated with MetS in some of the general and vulnerable populations at risk, but not all studies showed statistically significant associations despite the potential mechanisms supporting its benefit for metabolic health^11–14^. In addition, evidence from an observational study of non-metastatic colon cancer survivors in the United States showed some health benefits of dietary fiber intake including reduced mortality from cancer and any cause^15^. Yet, there is insufficient evidence for cancer survivors on the health benefits of dietary fiber intake in relation to MetS, which is an important clinical outcome for long-term health in cancer survivors^16–18^.

We therefore conducted the present study to evaluate the association between dietary fiber intake and MetS in adult cancer survivors from a nationally representative cross-sectional survey including sociodemographic, health behavior, health status, and dietary assessment.

## Results

### Characteristics of the study population

Sociodemographic, health behavior, clinical and nutritional status according to quintiles of dietary fiber intake in adult cancer survivors identified in the sixth and seventh KNHANES (2013–2018) are shown in Table 1. The mean dietary fiber intake was 28.1 g/day (standard error [SE], 0.54) and median (interquartile range, IQR) dietary fiber intake from the first quintile to the fifth quintile were 12.0 g/day (IQR: 9.0 to 14.0), 19.0 g/day (IQR: 17.0 to 20.0), 25.0 g/day (IQR: 23.0 to 27.0), 33.0 g/day (30.0 to 35.0), and 49.0 g/day (43.0 to 59.0), respectively. The adult cancer survivors in the highest quintile of dietary fiber intake were relatively young, with higher education attainment, and had healthy lifestyle (lower smoking and alcohol consumption rate and physically active). Across the quintiles of dietary fiber intake among adult cancer survivors, there was no statistically significant difference for metabolic risk profile. However, total energy intake was reported to be the highest in the adult cancer survivors of the fifth quintile of dietary fiber intake compared to the rest. Similar patterns were found for carbohydrate, fat, and protein intake.

**Table 1:** Characteristics of adult cancer survivors according to quintile of total dietary fiber intake in the Korea National Health and Nutrition Examination Survey VI and VII (2013–2018).

	Quintiles of dietary fiber intake					*p* value^b^
	Q1	Q2	Q3	Q4	Q5	
Dietary fiber intake g/day (median, IQR)	12.0 (9.0–14.0)	19.0 (17.0–20.0)	25.0 (23.0–27.0)	33.0 (30.0–35.0)	49.0 (43.0–59.0)	
No. of participants	271	267	255	248	260	
Metabolic Syndrome^a^	137 (50.6)	117 (43.8)	105 (41.2)	91 (36.7)	112 (43.1)	
**Age**						
< 65 years	115 (17.2)	144 (21.5)	134 (20.0)	132 (19.7)	145 (21.6)	0.0185
≥ 65 years	156 (24.7)	123 (19.5)	121 (19.2)	116 (18.4)	115 (18.2)	
**Sex**						
Male	80 (16.5)	94 (19.3)	99 (20.4)	101 (20.8)	112 (23.1)	0.1002
Female	191 (23.4)	173 (21.2)	156 (19.1)	147 (18.0)	148 (18.2)	
**Household income**^**c**^						
1Q	119 (31.6)	88 (23.3)	69 (18.3)	48 (12.7)	54 (14.1)	< 0.0001
2Q	65 (18.8)	66 (19.1)	76 (22.0)	72 (20.8)	67 (19.4)	
3Q	50 (16.6)	55 (18.2)	63 (20.9)	72 (23.8)	62 (20.5)	
4Q	37 (13.5)	58 (21.1)	46 (16.7)	56 (20.4)	78 (28.4)	
**Education level**						
Elementary school	139 (30.3)	106 (23.1)	85 (18.5)	64 (13.9)	65 (14.2)	< 0.0001
Middle school	31 (17.0)	37 (20.3)	40 (22.0)	29 (15.9)	45 (24.7)	
High school	58 (16.4)	59 (16.7)	82 (23.2)	88 (24.9)	67 (18.9)	
College/University	43 (14.1)	65 (21.2)	48 (15.7)	67 (21.9)	83 (27.1)	
**Cigarette smoking**						
Current smoker	23 (23.7)	22 (22.7)	20 (20.6)	18 (18.6)	14 (14.4)	0.4967
Past smoker	62 (17.0)	74 (20.3)	69 (19.0)	74 (20.3)	85 (23.4)	
Never smoker	186 (22.1)	171 (20.4)	166 (19.8)	156 (18.6)	161 (19.2)	
**Alcohol consumption**						
Non-drinker	63 (17.8)	67 (18.9)	63 (17.8)	73 (20.6)	89 (25.1)	0.0603
Drinker	208 (22.0)	200 (21.1)	192 (20.3)	175 (18.5)	171 (18.1)	
**Physical activity**						
Aerobic exercise	72 (15.3)	99 (21.0)	94 (20.0)	98 (20.8)	108 (22.9)	0.0333
Muscle-strengthening exercise	38 (13.0)	50 (17.1)	51 (17.4)	62 (21.2)	92 (31.4)	< 0.0001
Balance and flexibility exercise	54 (18.1)	66 (22.2)	52 (17.5)	53 (17.8)	73 (24.5)	0.4420
**Metabolic risk profile, mean (SE)**						
WC, cm	82.6 (0.6)	80.7 (0.8)	81.3 (0.5)	81.6 (0.7)	82.9 (0.7)	0.492
SBP, mmHg	123.9 (1.4)	119.8 (1.1)	122.3 (1.1)	123.7 (1.3)	121.3 (1.2)	0.7455
DBP, mmHg	74.5 (0.7)	75.1 (0.7)	75.4 (0.7)	75.4 (0.7)	74.7 (0.7)	0.803
HDL-C, mg/dL	49.9 (0.8)	51.7 (0.9)	50.5 (0.8)	49.3 (0.7)	50.7 (1.0)	0.8634
TG, mg/dL	139.6 (8.5)	125.5 (7.3)	123.7 (4.3)	127.6 (5.8)	127.9 (8.8)	0.4301
FSG, mg/dL	104.6 (1.8)	102.7 (1.4)	104.6 (2.4)	100.6 (1.3)	102.1 (1.2)	0.1381
Total energy intake, mean (SE), kcal/day	1282.2 (37.3)	1541.6 (32.3)	1760.1 (38.7)	1998.9 (43.9)	2458.3 (61.4)	< 0.0001
Carbohydrate intake, g/day	205 (5.1)	249.8 (4.7)	290.6 (5.5)	332.8 (6.8)	418.9 (9.6)	< 0.0001
Fat intake, g/day	42.5 (1.5)	52.9 (2.0)	62.5 (2.6)	69.8 (1.8)	91.2 (3.7)	< 0.0001
Protein intake, g/day	23.7 (1.5)	30.2 (1.4)	33.5 (1.5)	37.9 (1.6)	45.1 (2.3)	< 0.0001

Values above are presented as n (%) unless otherwise specified.

*Q* Quintile, *IQR* Interquartile range, *SE* Standard error, *WC* Waist circumference, *SBP* Systolic blood pressure, *DBP* Diastolic blood pressure, *HDL-C* High density lipoprotein cholesterol, *TG* Triglyceride, *FSG* Fasting serum glucose.

^a^Defined from the National Cholesterol Education Program Adult Treatment Panel III (NCEP-ATPIII) modified for the Asian population.

^b^Calculated from chi-square test for categorical variables and survey regression for continuous variables, respectively.

^c^Proxy for socioeconomic status (calculated from the sum of the income from the members of each household divided by the square root of the number of household members).

### Association between dietary fiber intake and MetS in adult cancer survivors

**Figure 1:**
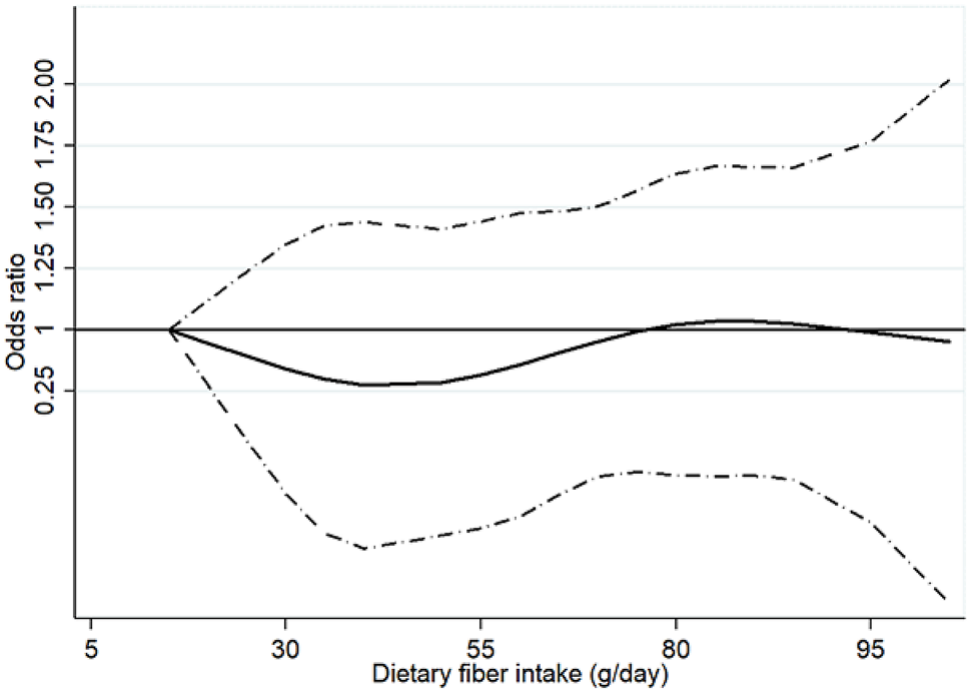
Relation between dietary fiber intake and metabolic syndrome in adult cancer survivors in the Korea National Health and Nutrition Examination Survey VI and VII (2013–2018), fitted with restricted cubic splines. The curves above are adjusted for sociodemographic (age, sex, education level, household income), health behavior (physical activity, cigarette smoking, alcohol consumption) and nutritional status (total energy intake).

In this cross-sectional study, multivariable logistic regression analyses showed a marginally inverse association between dietary fiber intake and MetS in adult cancer survivors. Compared to the lowest category of dietary fiber intake (the first quintile), the adjusted OR and 95% CI for MetS in the second, third, and fifth quintile were 0.93 (0.31–2.79), 0.91 (0.25–3.37), 0.66 (0.20–2.21), and 0.30 (0.09–1.00) for Model 1 (adjusted for age, sex, and physical activity), 0.87 (0.28–2.68), 0.81 (0.19–3.46), 0.59 (0.17–2.03), and 0.29 (0.09–0.92) for Model 2 (adjusted for household income, education level, cigarette smoking, alcohol consumption in addition to the variables included in Model 1), 0.84 (0.27–2.61), 0.77 (0.16–3.74), 0.55 (0.14–2.22), and 0.26 (0.05–1.39) for Model 3 (adjusted for variables included in Model 2 and total energy intake), respectively. *P*-value for linear trend across the quintiles of the dietary fiber intake in association with MetS for Model 1, Model 2, and Model 3 were 0.0001, 0.0004, and 0.0007, respectively (Table 2). Similar to the analysis of the association between dietary fiber intake as categorical values and MetS, the restricted cubic splines showed the marginally protective association of dietary fiber intake with MetS with confidence intervals exceeding the statistical significance level in adult cancer survivors (Fig. 1). The association between dietary fiber intake and MetS in cancer survivors did not significantly vary by the sociodemographic (age, sex, household income, education level), health behavior (cigarette smoking, alcohol consumption, physical activity) and nutritional status (total energy intake) for the lowest (the first quintile) versus highest (the fifth quintile) analyses (*p* value for > 0.05 interaction for all comparisons) (Table 3).

**Table 2:** Association of dietary fiber intake and metabolic syndrome in adult cancer survivors in the Korea National Health and Nutrition Examination Survey VI and VII (2013–2018).

	Quintiles of dietary fiber intake					*p* value for trend
	Q1 (n = 271)	Q2 (n = 267)	Q3 (n = 255)	Q4 (n = 248)	Q5 (n = 260)	
Dietary fiber intake g/day (median, IQR)	12.0 (9.0–14.0)	19.0 (17.0–20.0)	25.0 (23.0–27.0)	33.0 (30.0–35.0)	49.0 (43.0–59.0)	
No. of cases	137	117	105	91	112	
Model 1^a^ OR (95% CI)	1.00 (referent)	0.93 (0.31–2.79)	0.91 (0.25–3.37)	0.66 (0.20–2.21)	0.30 (0.09–1.00)	0.0001
Model 2^b^ OR (95% CI)	1.00 (referent)	0.87 (0.28–2.68)	0.81 (0.19–3.46)	0.59 (0.17–2.03)	0.29 (0.09–0.92)	0.0004
Model 3^c^ OR (95% CI)	1.00 (referent)	0.84 (0.27–2.61)	0.77 (0.16–3.74)	0.55 (0.14–2.22)	0.26 (0.05–1.39)	0.0007

OR (95% CI) presented above are weighted according to the national adult population of the KNHANES (2013–2018).

*Q* Quintile, *IQR* Interquartile range, *OR* Odds ratio, *CI* Confidence interval, *KNHANES* Korea National Health and Nutrition Examination Survey.

^a^Adjusted for age, sex, and physical activity.

^b^Adjusted for age, sex, physical activity, household income, education level, cigarette smoking, alcohol consumption.

^c^Adjusted for age, sex, physical activity, household income, education level, cigarette smoking, alcohol consumption, and total energy intake.

**Table 3:** Subgroup analysis for association of dietary fiber intake and metabolic syndrome in adult cancer survivors in the Korea National Health and Nutrition Examination Survey VI and VII (2013–2018).

	Dietary fiber intake		*p* value for interaction
	Lowest category of dietary fiber intake (Q1)	Highest category of dietary fiber intake (Q5)	
**Age, years**			
< 65 (n = 670)	1.00 (referent)	0.34 (0.06–2.21)	0.0627
≥ 65 (n = 631)	1.00 (referent)	0.26 (0.02–3.62)	
**Sex**			
Male (n = 486)	1.00 (referent)	0.007 (0.001–0.36)	0.0513
Female (n = 815)	1.00 (referent)	0.30 (0.03–2.78)	
**Household income**^**a**^			
Upper half (n = 578)	1.00 (referent)	0.57(0.05–7.00)	0.9323
Lower half (n = 723)	1.00 (referent)	0.13 (0.02–1.04)	
**Education level**			
< high school (n = 641)	1.00 (referent)	0.16 (0.01–2.33)	0.5962
≥ high school (n = 660)	1.00 (referent)	0.12 (0.01–1.28)	
**Cigarette smoking**			
Current/past smoker (n = 461)	1.00 (referent)	0.19 (0.01–2.72)	0.2512
Never smoker (n = 840)	1.00 (referent)	0.23 (0.03–1.77)	
**Alcohol consumption**			
Non-drinker (n = 355)	1.00 (referent)	0.45 (0.01–41.7)	0.6679
Drinker (n = 946)	1.00 (referent)	0.16 (0.02–1.09)	
**Physical activity**			
Physically active^b^ (n = 743)	1.00 (referent)	0.35 (0.05–2.22)	0.1876
Physically inactive^c^ (n = 558)	1.00 (referent)	0.11 (0.01–1.30)	
**Total energy intake**			
< 2000 kcal/day	1.00 (referent)	0.32 (0.04–2.37)	0.8382
≥ 2000 kcal/day	1.00 (referent)	0.30 (0.06–1.41)	

OR (95% CI) above are presented as odds ratio and 95% confidence intervals from multiple logistic regression adjusted for age, sex, household income, education level, cigarette smoking, alcohol consumption, physical activity, and total energy intake (except for the variable in the subgroup category) and all values presented above are weighted according to the national adult population of the KNHANES (2013–2018).

^a^Proxy for socioeconomic status (calculated from the sum of the income from the members of each household divided by the square root of the number of household members).

^b^Reported to engage in at least one of the following activities: aerobic, muscle-strengthening, balance and flexibility exercise.

^c^Reported to engage in none of the following activities: aerobic, muscle-strengthening, balance and flexibility exercise (does not exclude leisure-time activity).

*Q* Quintile, *KNHANES* Korea National Health and Nutrition Examination.

## Discussion

In the current study, we found a marginally protective association between high dietary fiber intake with MetS in community-dwelling adult cancer survivors. Compared to the lowest dietary fiber intake, the highest dietary fiber intake was associated with 74% lower odds of MetS that did not reach statistical significance after adjusting for potential confounders. However, the overall linear trend across the quintiles of dietary fiber intake in relation to the odds of MetS was observed.

Our results from this study are similar to those cross-sectional and cohort studies showing relationship between dietary fiber and MetS in both general population and vulnerable population at risk. A recent meta-analysis based on 11 cross-sectional and 3 cohort studies comprised of 26,403 total study subjects have shown that dietary fiber intake was inversely associated with MetS with lower risk comparing the highest with the lowest intake^5^. Pooled together, cross-sectional studies showed 30% lower odds and cohort studies showed 14% decreased risk of MetS in the highest consumption of dietary fiber compared to the lowest category. However, high heterogeneity and publication bias were found in the cross-sectional studies and the pooled relative risk from the cohort studies did not reach statistical significance. One of the notable studies conducted in Asia included in this meta-analysis was the study based on the Fukuoka Diabetes Registry (FDR) with 4,399 participants, which showed an inverse relationship of dietary fiber with MetS (OR: 0.92; 95% CI: 0.89–0.96)^19^. While the findings from our study and the FDR study is comparable, the definition of MetS in the FDR was harmonizing the metabolic syndrome^20^, which excludes the impaired fasting glucose as the components compared to the NCEP-ATP III definition. In addition, the study subjects of the FDR were limited to the participants with diabetes mellitus. Our study was focused on the association of dietary fiber intake with MetS in adult cancer survivors with limited statistical evidence supporting the protective association.

Only a few previous studies have investigated the health benefits of fiber intake in the high risk population including cancer survivors. In the prospective study of health professionals after diagnosis of non-metastatic colorectal cancer (CRC) in the U.S cohort, the Nurses’ Health Study (NHS) and Health Professionals Follow-up Study (HPFS) showed that high fiber intake was associated with lower CRC-specific and all-cause mortality supporting evidence for improved health outcome for high fiber consumption^15^. In addition, two studies from the NHS and HPFS, high dietary fiber intake was inversely associated with mortality among the survivors of myocardial infarction (MI)^21^. Taken together, these findings provide evidence that high dietary fiber intake is associated with better health outcomes in the vulnerable populations such as patients diagnosed with CRC or MI. However, the endpoint of these studies from the two large U.S cohorts were mortality instead of MetS. Our study investigated the health benefits of dietary fiber intake for MetS and found that high dietary fiber intake is marginally associated with lower odds of MetS.

High dietary fiber intake has been linked to improving components of MetS with potential mechanisms that support the beneficial role albeit these do not fully suggest that exact mechanisms could be directly applied to adult cancer survivors. Consumption of high fiber may reduce abdominal obesity through regulating energy homeostasis and altering gut microbiota^22,23^. High fiber intake is also reported to have cholesterol-lowering effect potentially attributable to reducing glycemic response after food consumption along with appetite control (i.e. inducing satiation and satiety)^24^. Previous observational studies consistently reported that high dietary fiber intake is inversely associated with the risk of type 2 diabetes mellitus and suggested that consumption of high dietary fiber may modulate inflammatory cytokines (e.g. interleukin-1 (IL-1), IL-12, and IL-18, tumor necrosis factor alpha (TNF-α), interferon gamma (IFNγ), and granulocyte–macrophage colony stimulating factor (GM-CSF)) and substantially improve glucose hemostasis^25–27^. Unlike the possible mechanisms that may partially explain the protective role of dietary fiber on MetS, mechanism behind consumption of high dietary fiber intake and reduced systolic and diastolic blood pressure is poorly understood. While these mechanisms are widely used to support the previous studies that reported protective association of high dietary fiber intake with MetS, the findings of our study showed that the association was not substantial, but rather marginal, possibly due to the limited statistical power for the adult cancer survivors identified in the KNHANES.

The most notable strength in this study includes assessment of dietary fiber intake in relation to MetS using a population-based sample of adult cancer survivors, which was understudied yet an important issue for clinical nutrition in cancer survivors. However, there are some limitations in this study that should be addressed when interpreting the results from the perspective of public health policy and clinical practice for adult cancer survivors. First, information on dietary fiber intake was obtained from the self-administered 24-h recall questionnaire in the KNHANES, which were not validated through consistent dietary records of the study participants. Thus, under or over estimation of dietary fiber intake in this study was inevitable due to the limited source of the dietary assessment. Considering the inevitable recall bias and other limitations of the 24-h recall approach for dietary fiber intake assessment, the future studies validating the results of this study with different dietary assessment methods are needed^28^. Second, we were not able to extensively consider other confounding variables that were not included in the KNHANES. Third, we were only able to conduct cross-sectional data analysis due to the study design of the KNHANES. Therefore, we were not able to determine whether the adult cancer survivors with high dietary fiber intake already had less odds of MetS or were less likely to develop newly diagnosed MetS in the follow-up period. Finally, the generalizability of the results found in this study might be limited because the sampling weight of the KNHANES was not originally designed to represent the entire population of cancer survivors in the Republic of Korea. Therefore, the weighted analyses conducted in accordance with the sampling design of the KNHANES could not fully reflect the entire cancer survivors from a statistical standpoint.

Evidence-based dietary guidelines for adult cancer survivors for the adequate range of daily dietary fiber intake for improving metabolic health needs further investigation as our study had limited statistical power. A marginally protective trend was observed for high dietary fiber intake and MetS up to approximately 43.0 g/day of dietary fiber intake in the restricted cubic splines with wide confidence intervals.

In conclusion, among the community-dwelling adult cancer survivors in the Republic of Korea, we observed marginally protective association of dietary fiber intake with MetS. As a part of the nutritional approaches to reduce the odds of MetS for adult cancer survivors, beneficial effects of increase in dietary fiber intake from variety of food consumption such as fruit, leafy vegetables, beans and whole grains should be carefully reviewed. Future investigations on the association of dietary fiber intake and MetS in adult cancer survivors should include well-designed observational studies or randomized clinical trials to update and support the evidence on the current guidelines on clinical nutrition for cancer survivors.

## Materials and methods

### Study population, design, and data collection

**Figure 2:**
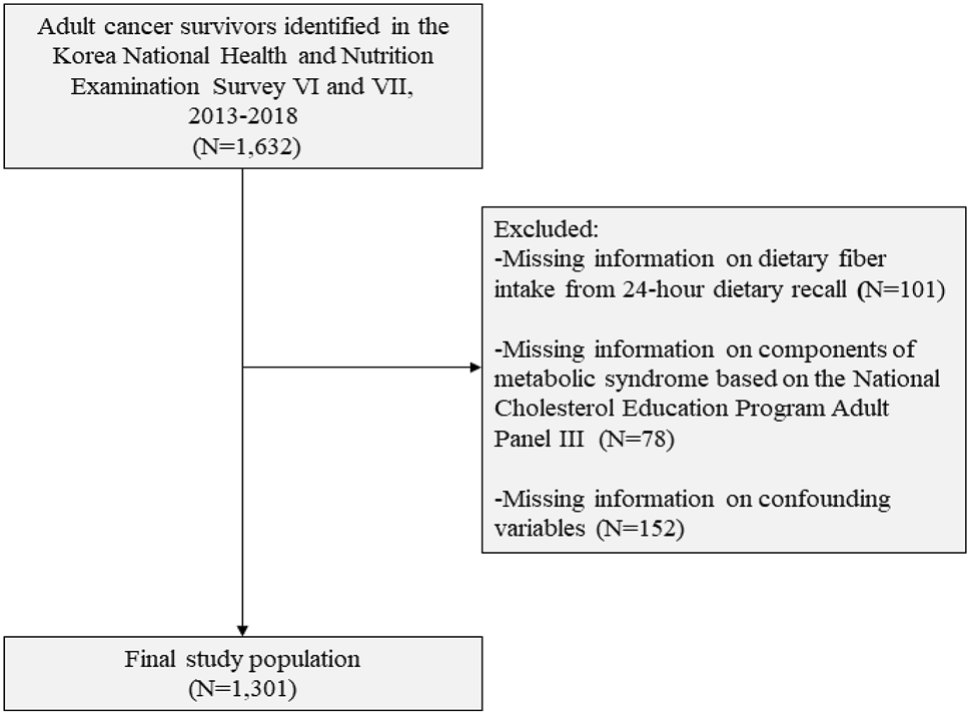
Study population flowchart diagram for adult cancer survivors in the Korea National Health and Nutrition Examination Survey VI and VII (2013–2018).

The study population in this study were community-dwelling cancer survivors who participated in the sixth and seventh Korea National Health and Nutrition Examination Survey (KNHANES VI-VII, 2013–2018), a nationally representative cross-sectional study consist of non-institutionalized individuals that initiated in 1998 by the Korea Disease Control and Prevention Agency (KDCA) in the Republic of Korea. Details of the KNHANES are available elsewhere^29^ and the KNHANES database had been used in various studies previously^30–34^. Among the participants of the sixth and seventh KNHANES, we identified 1632 adult (aged more than 20 years) cancer survivors from the self-reported questionnaire on the history of any type of cancer diagnosis. Of these adult cancer survivors in the dataset, we excluded those with missing information on dietary fiber intake information from the self-administered 24-h dietary recall (N = 101), missing information on the components of MetS (N = 78), and missing information on confounding variables (N = 152). The final study population included 1301 adult cancer survivors (Fig. 2).

### Assessment of dietary fiber intake

The self-administered 24-h recall questionnaire for daily dietary intake in the KNHANES was used to assess the dietary fiber intake (grams per day, g/day) among cancer survivors. Based on this information, dietary fiber intake was divided into quintiles from the lowest (the first quintile) to the highest (the fifth quintile), and the median and interquartile ratio (IQR) were obtained for each quintile. The validity of the self-administered 24-h recall questionnaire for dietary assessment has been described previously^35^. In addition, a previous study reported that 24-h recall method in the KNHANES had high correlation with the Food Frequency Questionnaire for usual portion size and consumption-day intake^36^.

### Definition of metabolic syndrome

Parts of the sixth and seventh KNHANES (2013–2018) consists of laboratory data, anthropometric measurements by trained medical staff and survey on clinical treatment for few diseases. Based on these data, we collected information on waist circumference (WC), systolic blood pressure (SBP), diastolic blood pressure (DBP), antihypertensive drug use, serum high density lipoprotein cholesterol (HDL-C), serum triglycerides (TG), lipid-lowering drug use, fasting serum glucose (FSG), and hypoglycemic agent or insulin treatment. We defined MetS according to the National Cholesterol Education Program Adult Treatment Panel III (NCEP-ATPIII) modified for the Asian population^37–39^. Therefore, cancer survivors in the KNHANES with at least three of the following components were defined as having MetS: (1) abdominal obesity (WC ≥ 90 cm for men and WC ≥ 85 cm for women, respectively); (2) elevated TG (serum TG ≥ 150 mg/dL or lipid-lowering drug use); (3) reduced HDL-C (serum HDL-C ≤ 40 mg/dL for men and ≤ 50 mg/dL for women, respectively); (4) elevated BP (SBP ≥ 130 mmHg or DBP ≥ 85 mmHg or with antihypertensive drug use); (5) elevated FSG (serum FSG ≥ 100 mg/dL or receiving hypoglycemic agent or insulin treatment). The modified definition of MetS in the KNHANES were used in the previous studies.

### Assessment of confounding variables

We obtained information on sociodemographic (age, sex, household income, and education level), health behavior (cigarette smoking, physical activity, and alcohol consumption), and dietary intake (total energy intake, carbohydrate intake, fat intake, and protein intake) from self-reported survey in the KNHANES. The household income was calculated from the summation of total income among household members divided by the square root of the number of household members. Physical activity was categorized into engaging in aerobic exercise, muscle-strengthening exercise, and balance and flexibility exercise in accordance to the recommended types of physical activity listed in the *Physical Activity Guidelines for Americans, 2nd edition* released in 2018^40^.

### Statistical analysis

To take complex, multistage, and probability sampling design of the KNHANES into account in all of the statistical analyses in this study, we used the appropriate procedure in the statistical software (e.g. “PROC SURVEYFREQ” and “svy” commands in SAS and STATA, respectively) for the results to represent the entire population of the Republic of Korea. Characteristics of the cancer survivors according to quintiles of dietary fiber intake were assessed using numbers (percentages) for categorical variables and means (standard error [SD]) for continuous variables with survey weights of the KNHANES taken into consideration. We used chi-squared test to compare the categorical variables and survey-weighted linear regression model to compute linear trend of the continuous variables, respectively. To estimate the odds of MetS according to quintiles of dietary fiber intake among cancer survivors, we used multiple logistic regression models and computed odds ratio (OR) and 95% confidence intervals (95% CI) using the lowest dietary fiber intake (the first quintile) as reference. First, we developed a minimally adjusted model (Model 1 adjusted for age, sex, and physical activity). In addition to Model 1, Model 2 was further adjusted for household income, education level, cigarette smoking, and alcohol consumption. Finally, Model 3 was adjusted for total energy intake with all other variables included in Model 2. To further assess the relation between dietary fiber intake and MetS among cancer survivors, we used restricted cubic splines of dietary fiber intake with five knots using multiple logistic regression adjusted for all of the variables included in Model 3. All analyses in this study were conducted using SAS software version 9.4 (SAS Institute., NC, USA) and STATA 14 (STATA Corp., College Station, TX, USA). *p* values less than 0.05 were considered statistically significant.

### Ethics statement

The protocols, conduct of the research, and data release for the sixth and seventh KNHANES (2013–2018) was approved by the KCDA Institutional Review Board (2013-12EXP-03-5C). All of the participants provided informed consent prior to participating in the KNHANES. This study was conducted in accordance to the guidelines of the Declaration of Helsinki-ethnical principles for medical research involving human subjects. The KNHANES dataset is publicly available for research purpose upon approval by the KDCA (www.kdca.go.kr).
